# Circ_0005918 Sponges miR-622 to Aggravate Intervertebral Disc Degeneration

**DOI:** 10.3389/fcell.2022.905213

**Published:** 2022-07-08

**Authors:** Yan Cui, Xintong Zhao, Yangang Wu

**Affiliations:** Department of Orthopaedics, the Hospital of Shunyi District, Beijing, China

**Keywords:** intervertebral disc degeneration, circular RNAs, circ_0005918, miR-622, cancers

## Abstract

Intervertebral discdegeneration (IDD) is the most common cause of lower back pain, but the exact molecular mechanism of IDD is still unknown. Recently, studies have shown that circular RNAs (circRNAs) regulate diverse biological procedures such as cell metastasis, growth, metabolism, migration, apoptosis, and invasion. We demonstrated that IL-1β and TNF-α induced circ_0005918 expression in the NP cell, and circ_0005918 was overexpressed in the IDD group compared with the control group. Moreover, the upregulated expression of circ_0005918 was associated with disc degeneration degree. The elevated expression of circ_0005918 promoted cell growth and ECM degradation, and it induced secretion of inflammatory cytokines including IL-1β, IL-6, and TNF-α. Moreover, we found that circ_0005918 sponged miR-622 in the NP cell. In addition, the exposure to IL-1β and TNF-α suppressed the expression of miR-622, which was downregulated in the IDD group compared with the control group. Furthermore, the downregulated expression of miR-622 was associated with disc degeneration degree. The expression level of miR-622 was negatively associated with circ_0005918 expression in the IDD group. In conclusion, circ_0005918 regulated cell growth, ECM degradation, and secretion of inflammatory cytokines by regulating miR-622 expression. These data suggested that circ_0005918 played important roles in the development of IDD via sponging miR-622.

## Introduction

Intervertebral disc degeneration (IDD) is a musculoskeletal disease, and is the most common cause of lower back pain, which affects a large portion of the population worldwide (Tan et al., 2018; Zhan et al., 2019; Zhao et al., 2019; Cao S. et al., 2021). IDD affects not only the quality of life but also causes economic and medical burdens to society (Chen W. et al., 2021; Li et al., 2021a; Tan et al., 2021). IDD is usually considered a natural disc aging procedure; however, a lot of patients demonstrate accelerated disc degeneration due to genetic and environmental factors (Chen et al., 2017; Wang et al., 2020; Sun et al., 2021; Zhan et al., 2021). The etiology and molecular mechanism of IDD are complicated and increasingly, evidence has shown that gender, age, inflammation, and behavioral influences are associated with the risk of IDD (Kraus et al., 2019; Jing and Liu, 2021; Tan et al., 2021). The exact molecular mechanism of IDD is still unknown. It is urgent to elucidate the underlying pathogenesis and find novel treatment strategies for IDD.

Circular RNAs (circRNAs), a new type of non-coding RNA, consist of exon transcripts and exert crucial roles in regulating the downstream gene expression (Liang et al., 2019; Ma et al., 2020; Ding et al., 2021; Yang et al., 2021). Increasing evidence has indicated that circRNAs regulate diverse cell biological procedures such as cell metastasis, growth, metabolism, migration, apoptosis, and invasion (Tian et al., 2019; Liu et al., 2020; Nie et al., 2020; Pu et al., 2020; Zhou et al., 2020). Recent studies have suggested that circRNAs participated in many diseases including congenital diseases, metabolic diseases, infection, and cancer (Li et al., 2019; Li et al., 2020a; Li et al., 2020b; Li et al., 2020c; Zhao et al., 2021). CircRNAs are involved in various orthopedic diseases such as osteoarthritis, femoral head necrosis, scoliosis, osteosarcoma, and also IDD (Guo et al., 2020a; Yan et al., 2020; Zhang et al., 2021a; Hao et al., 2021). For example, Hu et al. (2022) demonstrated that circ_0022382 suppressed IDD progression by modulating TGF-beta3/miR-4726-5p expression. Wang et al. (2021) showed that circARL15 played crucial roles in IDD development by sponging the miR-431-5p/DISC1 axis. Recently Guo et al. (2021) performed microarray hybridization and qRT-PCR assay to study differentially expressed circRNAs and identified that the expression of circ_0005918 was upregulated in the IDD group. However, its function in IDD remains unknown.

In our study, we studied the expression of circ_0005918 in IDD tissues and found that IL-1β and TNF-α induced circ_0005918 expression in the NP cell, and circ_0005918 was overexpressed in the IDD group compared with the control group. The elevated expression of circ_0005918 promoted cell growth and ECM degradation, and induced secretion of inflammatory cytokines including IL-1β, IL-6, and TNF-α.

## Materials and Methods

### Tissue Specimens

Our study was approved by the Clinical Ethics Committee of Shunyi Hospital, Beijing, China, and we followed the rules of the Helsinki Declaration. Participants gave written informed consent. Intervertebral disc specimens were collected from scoliosis patients who were undergoing osteotomy and spinal fusion as controls and intervertebral disc specimens from IDD patients who underwent surgery were collected in our hospital.

### Cell Culture and Cell Transfection

Eight NP samples were obtained from IDD that were used for isolating and culturing NP cells. The human NP cells were cultured according to previous references (Gao et al., 2020; Chen Y. et al., 2021). The NP cells were then passaged two times to apply for other experiments. Cell transfection was performed using Lipofectamine 2000 (Invitrogen). pcDNA3.1-circ_0005918, miR-622 mimic, and an empty vector (used as a negative control) were purchased from Invitrogen.

### Real-Time PCR

Total RNA was isolated from the NP samples or cells using a Trizol kit (Beyotime Biotechnology, China). The cDNA synthesis reagent (Sangon, Shanghai, China) was used for cDNA synthesis. The level of circ_0005918 and miR-622 was detected by real-time qRT-PCR using SYBR green (Applied Biosystems, Thermo) on the ABI 7500 system following the manufacturer’s protocols. The primer sequences were shown as follows: human circ_0005918 forward, 5′-AAA​GCT​ACC​CAC​GCA​AGG​AA-3′ and reverse, 5′-CTT​TGT​CAA​CTG​GTC​CAC​ACA​C-3'; miR-622 forward, 5′-ATCCC AGGGA GACAG AGATC GAGG-3′ and reverse, 5′-AAGCT TGGTG GTGGA CTTTT GGTTGT-3′; GAPHD: forward, 5′-GCACC GTCAA GGCTG AGAAC-3′ and reverse, 5′-TGGTG AAGAC GCCAG TGGA-3′; U6: forward, 5′-GGTGA AGCAG GCGTC GGAGG-3′ and reverse, 5′-GAGGG CAATG CCAGC CCCAG-3′. The relative level of these genes was determined by 2 ^−ΔΔCt^, and GAPDH was performed as an internal reference for mRNA and circRNA and U6 as an internal reference for miRNA.

### Cell Growth and ELISA

The NP cells were cultured in a 96-well culture dish at the density of 2*10^3^ per dish. The cell proliferation rate was measured for 0, 24, 48, and 72h, and 10 μL CCK-8 reagent was added to each dish for incubation for 2 h. The absorbance at 450 nm was detected using a microplate reader. The concentrations of IL-6, TNF-α, and IL-1β in the cell supernatants were detected using an ELISA kit following the kit’s instructions (Boste, China).

### Western Blot Analysis

Total protein from samples or cells was extracted with RIPA buffer. The concentration of protein was analyzed with a BCA protein assay. An equivalent quantity of protein was resolved on 12% SDS-PAGE (sodium dodecyl sulfate-polyacrylamide gel electrophoresis) and then transferred to the PVDF membrane (Bio-Rad). After blocking with bovine serum, the PVDF membrane was incubated with anti-GAPDH or anti-MMP9 overnight at 4°C. The antibody binding was measured with peroxidase-conjugated IgG and visualized using ECL chemiluminescent system (Pierce Biotechnology, IL) following the manufacturer’s description.

### Luciferase Assay

The sequences of circ_0005918 containing miR-622 complementary sites were cloned into the pGL3 luciferase vector, which was called circ_0005918-WT or circ_0005918-mut. The cells were cultured in the 96-well plate and co-transfected with miR-622 mimic or control and circ_0005918-WT or circ_0005918-mut. After incubation for 48 h, luciferase activity was analyzed by a dual-luciferase reporter kit (Promega, WI, United States).

### Statistical Analysis

Statistical assay was carried out by SPSS 20.0, and data between two groups were detected utilizing the Student’s test. *p*-value <0.05 was considered significantly different.

## Results

### Exposure to IL-1β and TNF-α Induced circ_0005918 Expression

**FIGURE 1:**
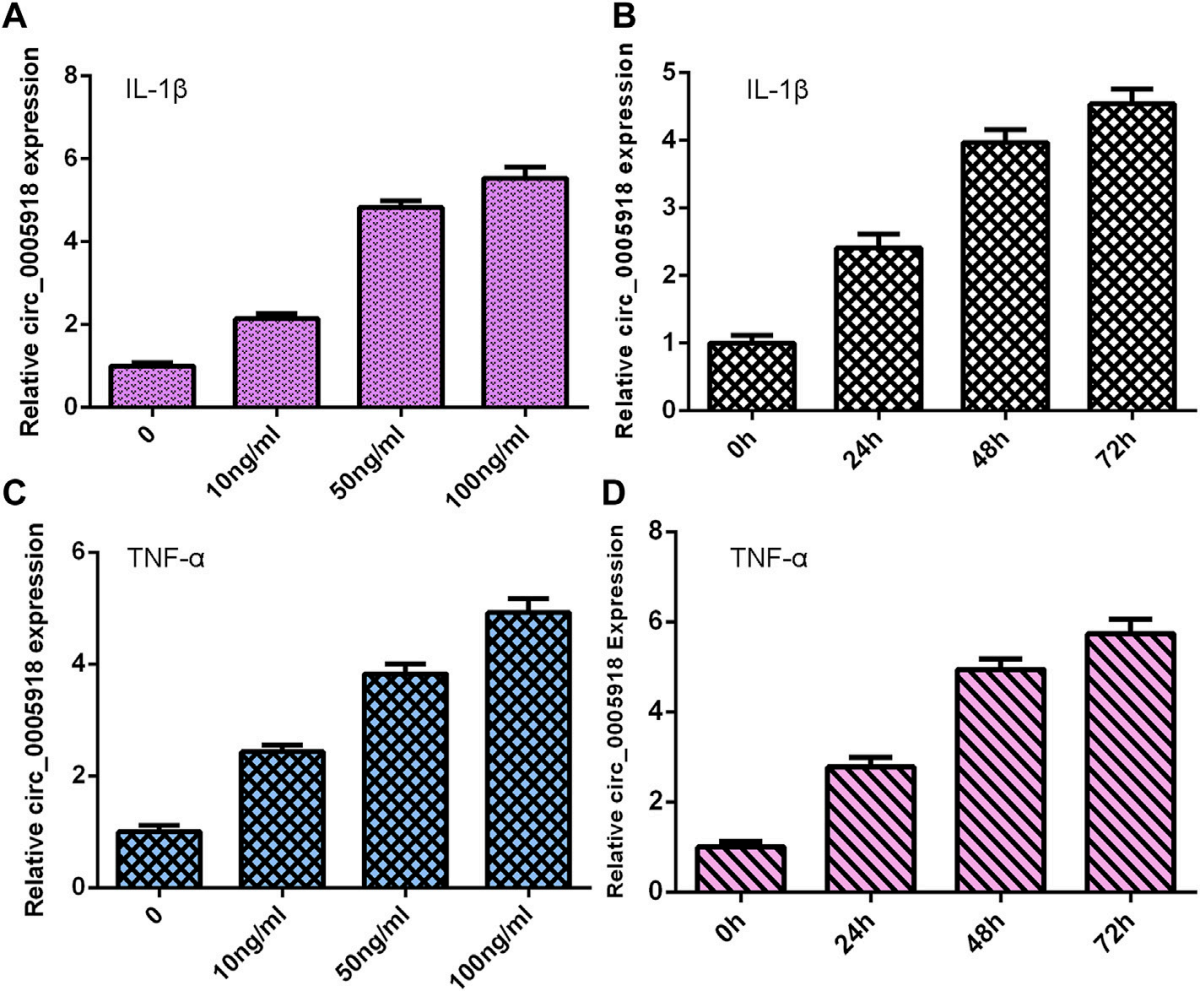
Exposure to IL-1β and TNF-α induced circ_0005918 expression. **(A)** Expression of circ_0005918 was measured by qRT-PCR assay. **(B)** IL-1β induced circ_0005918 expression in a time-dependent manner. **(C)** The level of circ_0005918 was detected via qRT-PCR assay. **(D)** TNF-α induced circ_0005918 expression in a time-dependent manner.

NP cells were treated with different doses of IL-1β and TNF-α for 48 h, and the expression of circ_0005918 was detected via qRT-PCR. As shown in Figures 1A,C, circ_0005918 expression was upregulated in the IL-1β and TNF-α groups compared with the control groups. Moreover, we showed that IL-1β and TNF-α induced circ_0005918 expression in a time-dependent manner (Figures 1B,D).

### Upregulated circ_0005918 Levels Were Associated With IDD

**FIGURE 2:**
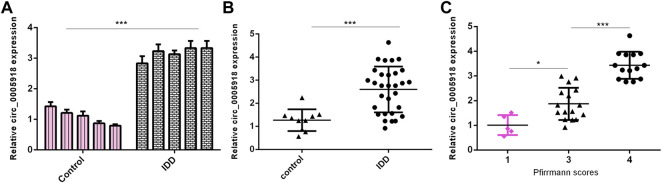
Upregulated circ_0005918 levels were associated with IDD. **(A)** The circ_0005918 level was upregulated 5 NP cells from IDD compared to control. **(B)** Circ_0005918 was overexpressed in the IDD group compared to the control group. **(C)** The upregulated expression of circ_0005918 was associated with disc degeneration levels. *p < 0.05 and ***p < 0.001.

Then, we measured circ_0005918 expression in the IVD samples. First, we showed that the circ_0005918 level was upregulated in NP cells from the IDD group compared with the control group (Figure 2A). Then, circ_0005918 was overexpressed in the IDD group compared with the control group (Figure 2B). Moreover, the upregulated expression of circ_0005918 was associated with disc degeneration degree (Figure 2C).

### Circ_0005918 Induced Cell Growth and ECM Degradation

**FIGURE 3:**
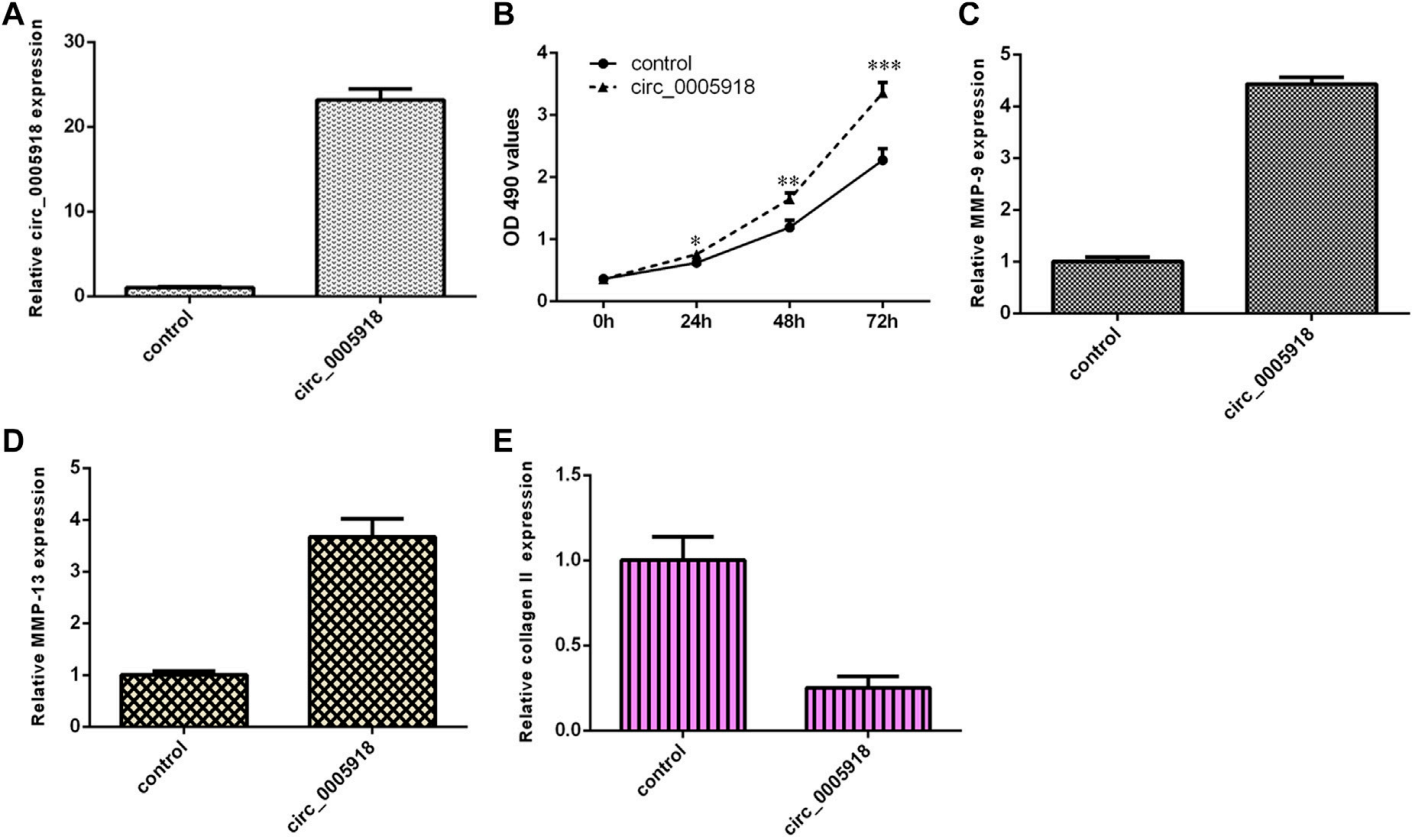
Circ_0005918 induced cell growth and ECM degradation. **(A)** The expression of circ_0005918 was measured by qRT-PCR assay. **(B)** The elevated expression of circ_0005918 promoted cell growth in the NP cell. **(C)** The overexpression of circ_0005918 promoted MMP-9 expressions in the NP cell. **(D)** The ectopic expression of circ_0005918 induced MMP-13 expression in the NP cell. **(E)** The overexpression of circ_0005918 suppressed collagen II expression in the NP cell. *p < 0.05, **p < 0.01, ***p < 0.001.

Circ_0005918 was remarkably overexpressed in the NP cell after being transfected with pcDNA-circ_0005918 (Figure 3A). The elevated expression of circ_0005918 promoted cell growth in the NP cell (Figure 3B). The overexpression of circ_0005918 promoted MMP-9 expression in the NP cell (Figure 3C). Ectopic expression of circ_0005918 induced MMP-13 expression in the NP cell (Figure 3D). However, the overexpression of circ_0005918 suppressed collagen II expression in the NP cell (Figure 3E).

### Circ_0005918 Induced Secretion of Inflammatory Cytokines

**FIGURE 4:**
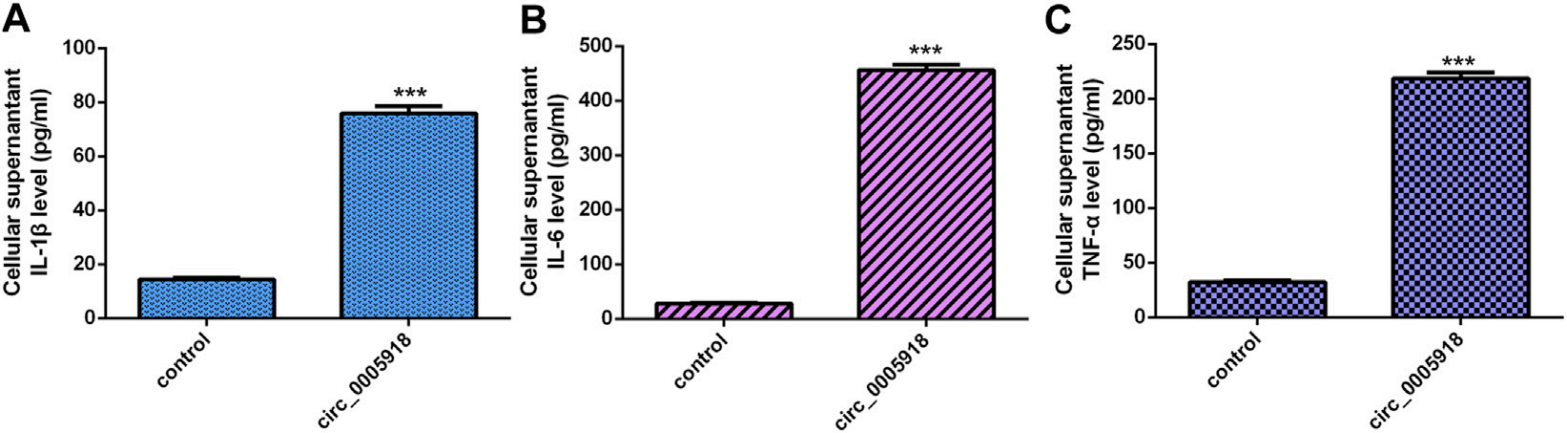
Circ_0005918 induced inflammatory cytokines secretion. **(A)** The ectopic expression of circ_0005918 promoted IL-1β expression. **(B)** The level of IL-6 in the cell supernatants was measured by ELISA. **(C)** The level of TNF-α in the cell supernatants was measured by ELISA. ***p < 0.001.

We showed that the ectopic expression of circ_0005918 promoted the secretion of three inflammatory cytokines including IL-1β (Figure 4A), IL-6 (Figure 4B), and TNF-α (Figure 4C).

### Circ_0005918 Regulated miR-622 Expression in NP Cell

**FIGURE 5:**
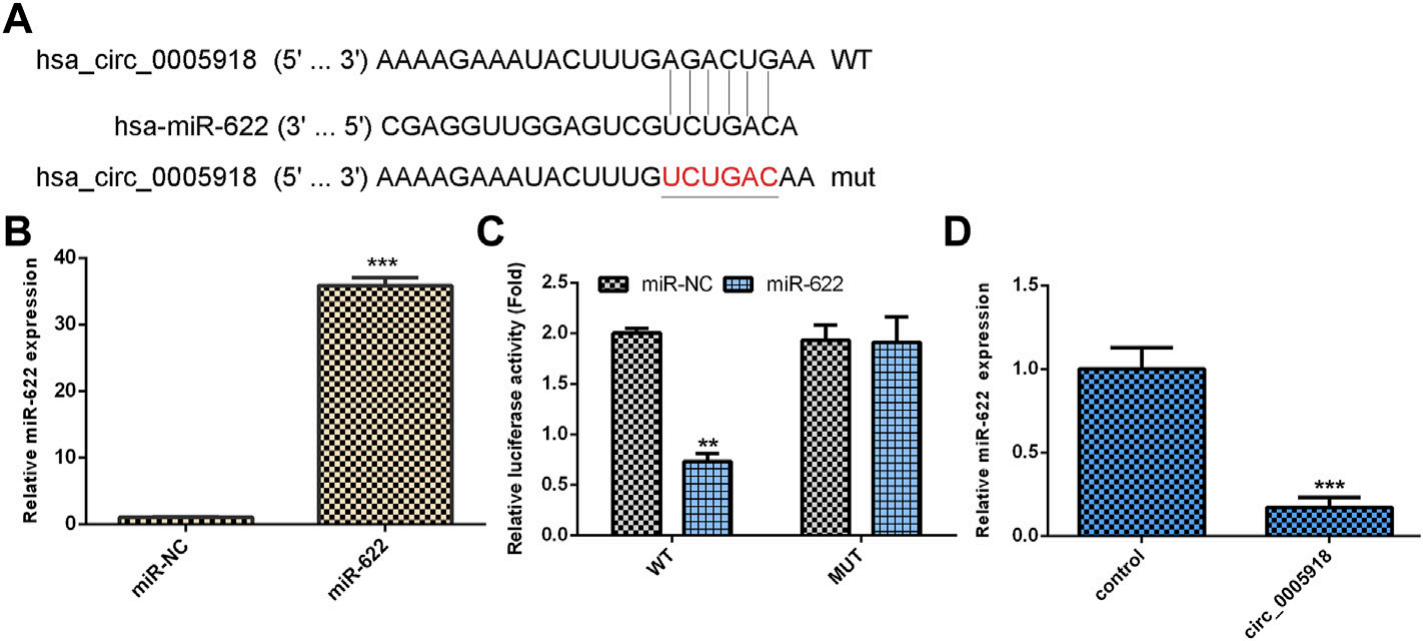
Circ_0005918 regulated miR-622 expression in NP cell. **(A)** By using Circular RNA Interactome software, it shows that circ_0005918 may sponge miR-622 expression. **(B)** The expression of miR-622 was measured by qRT-PCR assay. **(C)** The luciferase reporter indicated the ectopic expression of miR-622 suppressed the luciferase reporter value of circ_0005918 wild reporter vector but did not change the value of the mutant reporter. **(D)** The overexpression of circ_0005918 inhibited miR-622 expression. **p < 0.01, ***p < 0.001.

By using Circular RNA Interactome software, we showed that circ_0005918 may serve as a miR-622 sponge (Figure 5A). MiR-622 was significantly upregulated in the miR-622 mimic group compared with the control group (Figure 5B). The luciferase reporter indicated ectopic expression of miR-622 suppressed the luciferase reporter value of circ_0005918 wild reporter vector but did not change the value of the mutant reporter (Figure 5C). The overexpression of circ_0005918 inhibited miR-622 expression (Figure 5D).

### Exposure to IL-1β and TNF-α Suppressed miR-622 Expression

**FIGURE 6:**
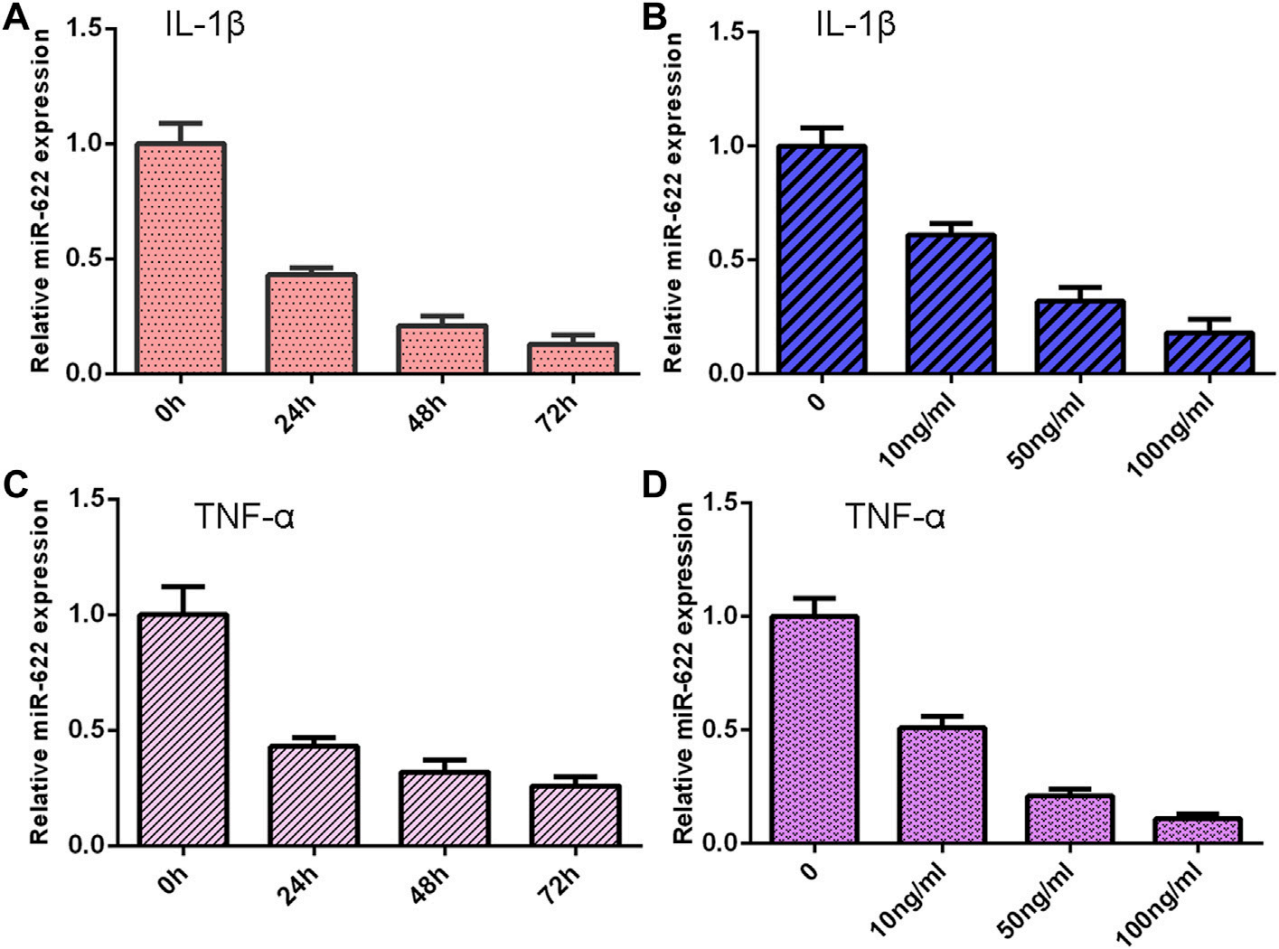
Exposure to IL-1β and TNF-α suppressed miR-622 expression. **(A)** The expression of miR-622 was measured by qRT-PCR assay. **(B)** IL-1β suppressed miR-622 expression in a time-dependent manner. **(C)** The level of miR-622 was detected via qRT-PCR assay. **(D)** TNF-α inhibited miR-622 expression in a time-dependent manner.

The NP cells were treated with different doses of IL-1β and TNF-α for 48 h, and the expression of miR-622 was detected via qRT-PCR. As shown in Figures 6A,C, the miR-622 expression was downregulated in the IL-1β and TNF-α groups compared with the control group. Moreover, we showed that IL-1β and TNF-α induced miR-622 expression in a time-dependent manner (Figures 6B,D).

### Downregulated miR-622 Level Was Associated With Degeneration Level in IDD

**FIGURE 7:**
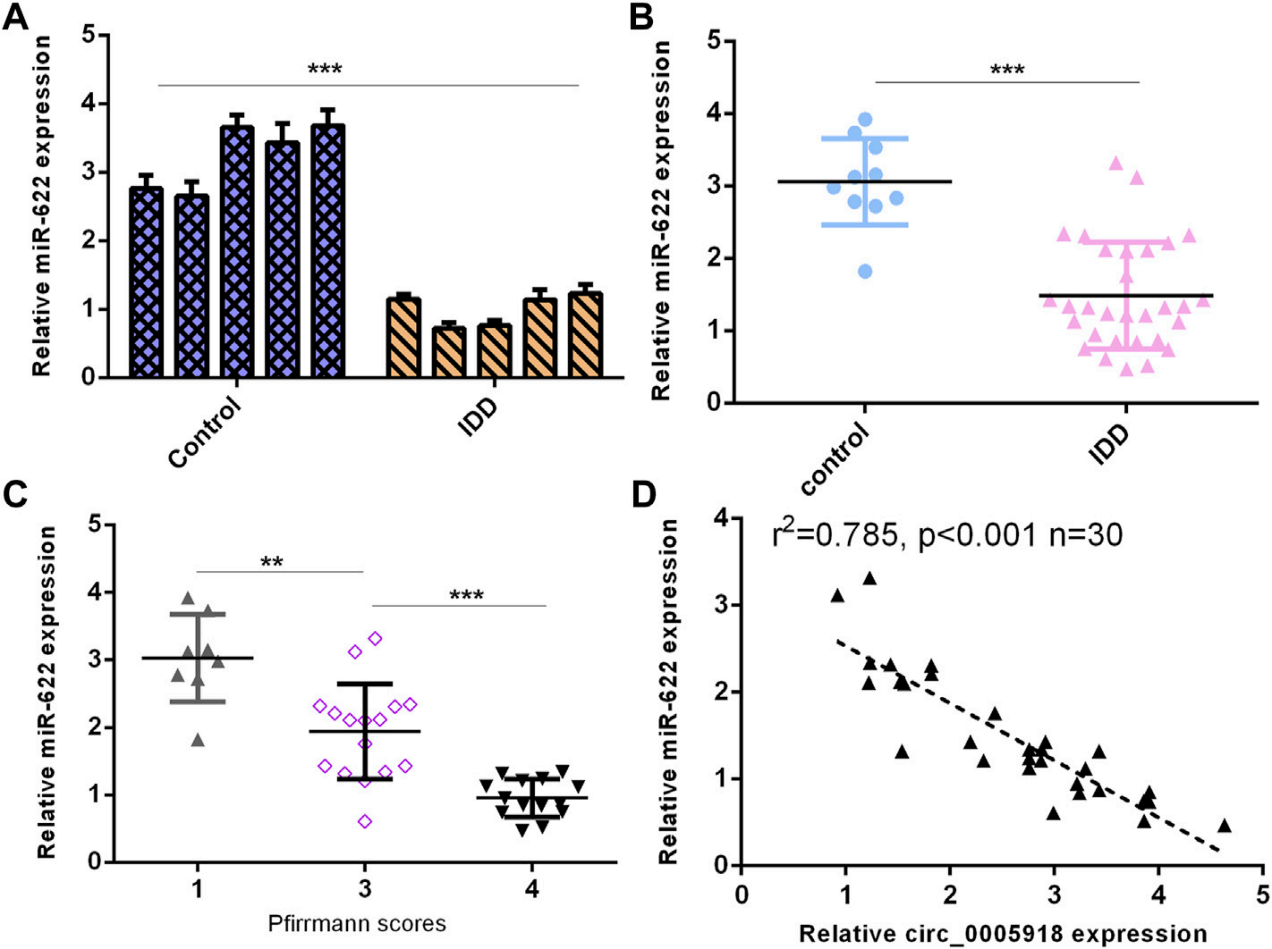
Downregulated miR-622 levels were associated with IDD. **(A)** miR-622 level was downregulated 5 NP cells from IDD compared to control. **(B)** miR-622 was lower in the IDD group than the control group. **(C)** The downregulated expression of miR-622 was associated with disc degeneration levels. **(D)** The level of miR-622 was negatively associated with circ_0005918 expression in the IDD group. **p < 0.01, ***p < 0.001.

Then, we detected miR-622 expression in the IVD samples. First, we showed that the miR-622 level was downregulated by 5 NP cells from IDD compared with the control group (Figure 7A). Then, the miR-622 expression was lower in the IDD group compared with that the control group (Figure 7B). Moreover, the downregulated expression of miR-622 was associated with disc degeneration levels (Figure 7C). The level of miR-622 was negatively associated with circ_0005918 expression in the IDD group (Figure 7D).

### Circ_0005918 Regulated Cell Growth, ECM Degradation Through Regulating miR-622

**FIGURE 8:**
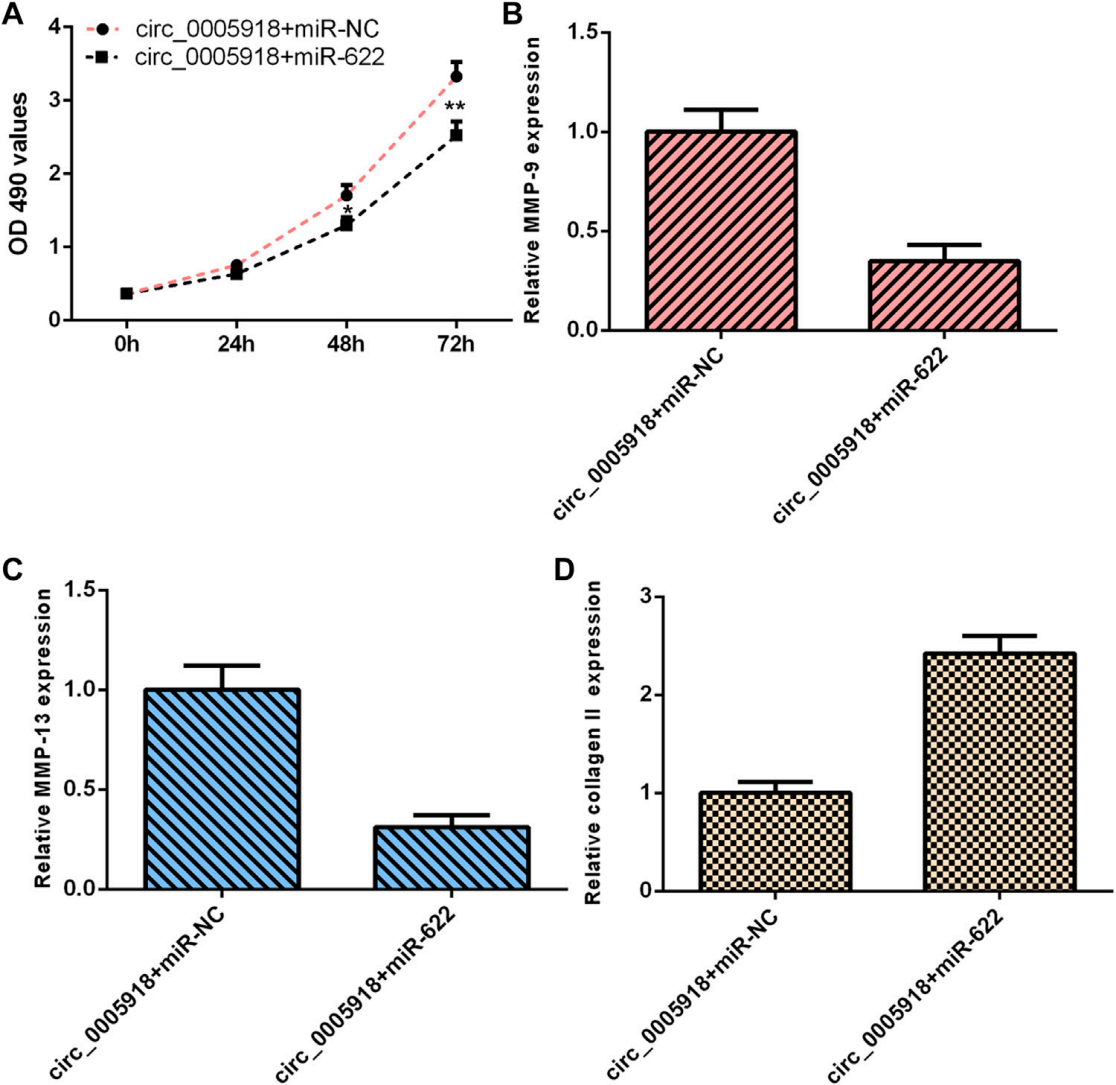
Circ_0005918 regulated the cell growth and ECM degradation by regulating miR-622. **(A)** Cell proliferation was detected by CCK-8 assay. **(B)** The ectopic expression of miR-622 suppressed MMP-9 expression in the circ_0005918-overexpressing NP cell. **(C)** The level of MMP-13 was measured by qRT-PCR analysis. **(D)** The level of collagen II was measured by qRT-PCR analysis. *p < 0.05, **p < 0.01.

As circ_0005918 sponged miR-622, we next studied whether circ_0005918 regulated cell function by modulating miR-622. The overexpression of circ_0005918 induced cell proliferation in NP cells. However, miR-622 mimic decreased this function (Figure 8A). The ectopic expression of miR-622 suppressed the expression of MMP-9 (Figure 8B) and MMP-13 (Figure 8C), and enhanced the expression of collagen II (Figure 8D) in the circ_0005918-overexpressing NP cell.

### Circ_0005918 Regulated Inflammatory Cytokines Secretion Through Regulating miR-622

**FIGURE 9:**
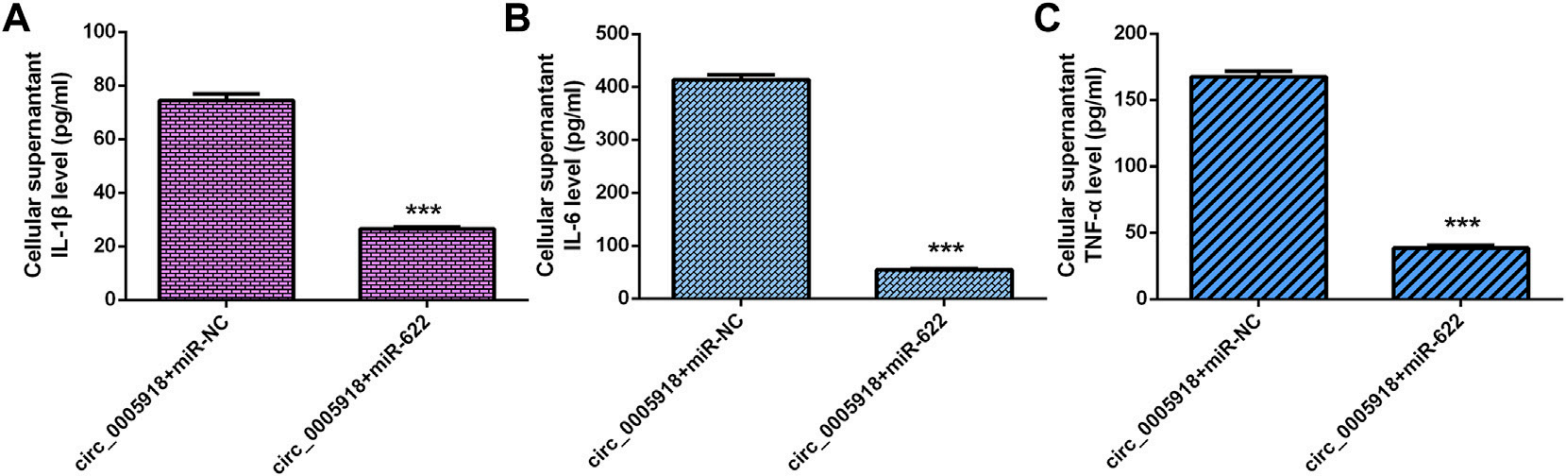
Circ_0005918 regulated the inflammatory cytokines secretion by regulating miR-622. **(A)** Elevated expression of circ_0005918 induced IL-1β expression, while miR-622 mimic suppressed this function in the NP cell. **(B)** The level of IL-6 in the cell supernatants was measured by ELISA. **(C)** The level of TNF-α in the cell supernatants was measured by ELISA. ***p < 0.001.

The elevated expression of circ_0005918 induced the secretion of 3 inflammatory cytokines including IL-1β (Figure 9A), IL-6 (Figure 9B), and TNF-α (Figure 9C), while miR-622 mimic suppressed this function in the NP cell.

## Discussion

In our study, we demonstrated that IL-1β and TNF-α induced circ_0005918 expression in the NP cell and circ_0005918 was overexpressed in the IDD group compared with the control group. Moreover, the upregulated expression of circ_0005918 was associated with disc degeneration levels. The elevated expression of circ_0005918 promoted cell growth, ECM degradation, and induced secretion of inflammatory cytokines, including IL-1β, IL-6, and TNF-α. Moreover, we found that circ_0005918 sponged miR-622 in the NP cell. In addition, the exposure to IL-1β and TNF-α suppressed miR-622 expression, and miR-622 was downregulated in the IDD group compared with the control group. Furthermore, the downregulated expression of miR-622 was associated with disc degeneration levels. The level of miR-622 was negatively associated with circ_0005918 expression in the IDD group. Circ_0005918 regulated cell growth, ECM degradation, and inflammatory cytokines secretion by regulating the miR-622 expression. These data suggested that circ_0005918 plays important roles in the development of IDD via sponging miR-622.

Growing evidence has shown that circRNAs play important roles in the development of IDD (Guo et al., 2020a; Guo et al., 2020b; Cui and Zhang, 2020). For instance, Huang et al. (2021a) demonstrated that circSPG21 decreased IDD development by regulating the miR-1197/ATP1B3 axis. Zhang et al. 2021b) found that circSNHG5 protected cartilage endplate degradation by modulating the miR-495-3p/CITED2 axis. Wang et al. (2021) showed that circARL15 was decreased in the IDD group and miR-431-5p, directly bound to DISC1 and circARL15. The elevated expression of circARL15 suppressed NP cell apoptosis but induced NP cell growth by regulating the miR-431-5p/DISC1 axis. Zhang et al. (2021c) demonstrated that circular RNA ITCH induced ECM degradation by promoting the Wnt/beta-catenin pathway in the development of IDD. Huang et al. (2021b) found that circPKNOX1 regulated IDD progression by modulating the miR-370-3p/KIAA0355 axis. Recently, Guo et al. (2021) performed microarray hybridization and qRT-PCR assay to study differentially expressed circRNA. They identified that the expression of circ_0005918 was upregulated in the IDD group. However, the function of circ_0005918 remains unknown. Therefore, we chose circ_0005918 to study and showed that circ_0005918 was overexpressed in the IDD group compared with the control group. Moreover, the upregulated expression of circ_0005918 was associated with disc degeneration degree. The elevated expression of circ_0005918 promoted cell growth, ECM degradation, and secretion of inflammatory cytokines including IL-1β, IL-6, and TNF-α.

Previous studies demonstrated that miRNAs are involved in the development of IDD. For example, Cao J. et al. (2021) showed that miR-200c-3p inhibited IDD development by regulating the RAP2C/ERK pathway. Lei et al. (2021) showed that miR-25 inhibited NP cell apoptosis by regulating SUMO2 in the IDD. Du et al. (2021) demonstrated that IL-1β induced miR-133a-5p expression to promote IDD development through regulating FBXO6. Rescuing FBXO6 and inhibiting the expression of miR-133a-5p may be potential strategies for IDD treatment. Recently, studies have shown that circRNAs might play their roles via sponging miRNAs. For instance, Li Y. et al. (2021) demonstrated that circ_0040039 induced IDD progression by modulating the MiR-874-3p/ESR1 pathway axis. Xiang et al. (2020) showed that downregulation of circCOG8 regulated the compression-induced IDD development by sponging the miR-182-5p/FOXO3 axis. In our study, by using Circular RNA Interactome software, we showed that circ_0005918 may sponge miR-622. The luciferase reporter indicated that ectopic expression of miR-622 suppressed the luciferase reporter value of circ_0005918 wild reporter vector but did not change the value of the mutant reporter. In addition, exposure to IL-1β and TNF-α suppressed miR-622 expression, and miR-622 was downregulated in the IDD group compared with the control group. Furthermore, the downregulated expression of miR-622 was associated with disc degeneration degree. The level of miR-622 was negatively associated with circ_0005918 expression in the IDD group. To conclude, circ_0005918 regulated cell growth, ECM degradation, and inflammatory cytokines secretion by regulating miR-622 expression.

In conclusion, our data demonstrated that IL-1β and TNF-α induced circ_0005918 expression in the NP cell. Circ_0005918 was overexpressed in IDD, and was associated with disc degeneration degree. Circ_0005918 regulated cell growth, ECM degradation, and inflammatory cytokines secretion by regulating miR-622 expression. We suggested that circ_0005918 plays important roles in the development of IDD via sponging miR-622.

## Data Availability

The original contributions presented in the study are included in the article/Supplementary Material; further inquiries can be directed to the corresponding author.
